# Oxazolidinones in the Clinical Management of Staphylococcal Infection—Linezolid or Tedizolid? A review on behalf of the European Society of Clinical Microbiology and Infectious Diseases (ESCMID) Study Group for Staphylococci and Staphylococcal Diseases (ESGS)

**DOI:** 10.1093/jac/dkag323

**Published:** 2026-10-12

**Authors:** Liana Macpherson, Anne-Grete Martson, Jose L Del Pozo, Cindy Zheng, Anna L Goodman

**Affiliations:** Department of Infection, Guy’s and St Thomas’ NHS Foundation Trust, London, UK; European Society of Clinical Microbiology and Infectious Diseases (ESCMID) Study Group for Staphylococci and Staphylococcal Diseases (ESGS); European Society of Clinical Microbiology and Infectious Diseases (ESCMID) Study Group for Staphylococci and Staphylococcal Diseases (ESGS); Leiden Academic Centre for Drug Research, Leiden University, Leiden, The Netherlands; European Society of Clinical Microbiology and Infectious Diseases (ESCMID) Study Group for Staphylococci and Staphylococcal Diseases (ESGS); Department of Infectious Diseases and Clinical Microbiology, Clínica Universidad de Navarra, Pamplona, Spain; IdiSNA, Navarra Institute for Health Research, Navarra, Spain; Department of Infection, Guy’s and St Thomas’ NHS Foundation Trust, London, UK; Department of Infection, Guy’s and St Thomas’ NHS Foundation Trust, London, UK; European Society of Clinical Microbiology and Infectious Diseases (ESCMID) Study Group for Staphylococci and Staphylococcal Diseases (ESGS); Innovative Clinical Trials Unit, Institute of Clinical Trials and Methodology, University College London, London, UK; Department of Infection, King’s College London, London, UK

## Abstract

There is limited information in the existing literature regarding the choice of oxazolidinone in clinical practice to treat infections caused by staphylococcal species. As members of the European Society of Clinical Microbiology and Infectious Diseases (ESCMID) Study Group for Staphylococci and Staphylococcal Diseases (ESGS) we have collaborated and written this narrative review aimed at clinicians considering these agents in practice. The review focuses on two medications, linezolid and tedizolid, with a comprehensive description of pharmacology aspects, adverse effects, drug interactions and contraindications, and we investigate the potential for treatment optimization. We anticipate that this article will provide support for those considering utilization of linezolid and tedizolid in clinical practice to treat staphylococcal species.

## Introduction

There is limited information in the existing literature regarding the choice of oxazolidinone in clinical practice to treat infections caused by staphylococcal species. As members of the ESCMID Study Group for Staphylococci and Staphylococcal Diseases (ESGS) we have collaborated and written this narrative review aimed to support clinicians considering these agents in practice.

Tedizolid and linezolid are oxazolidinone antibiotics that share a similar mechanism of action but have differences in potency and pharmacokinetics. Both are effective against Gram-positive organisms including resistant pathogens such as MRSA, VRE and some strains of CoNS. Linezolid is licensed for use in the UK by the Medicines and Healthcare products Regulatory Agency (MHRA) to treat a range of infections including nosocomial and community-acquired pneumonia and complicated skin and soft tissue infections. Tedizolid is approved for use to treat acute bacterial skin and skin structure infections (ABSSSIs). These agents are commonly selected for their effectiveness against resistant pathogens, tissue penetration and high bioavailability with oral administration, but their use is limited by toxicity and interactions with other medications. The most used agent is linezolid, with increasing use of tedizolid. The development of additional oxazolidinones has been limited by pharmacokinetic or toxicity concerns. Linezolid, developed by Upjohn Laboratories (now Pfizer) in 1996, was the first oxazolidinone class antibiotic to be approved by the US FDA in April 2000; subsequently tedizolid was approved by the FDA in 2014, and by the EMA in 2015. Other antibiotics in this group in different stages of development are shown in Table 1; however, as these are not currently used they are not included in this review.^1^

**Table 1.** Oxazolidinone antibiotics licensed, approved or in development

Agent	Developmental stage	IV, oral (PO) and dosing schedule
Linezolid	Licensed	PO/IV (twice daily)
Tedizolid	Licensed	PO/IV (once daily)
Contezolid (MRX-I)	Approved in China	PO (twice daily)
Delpazolid	Phase 2 trials	PO (once/twice daily)
Eperezolid	No longer in development (linezolid found to have superior PK/PD)	PO/IV (four times daily)
Posizolid	No longer in development (withdrawn due to hepatic and haematological toxicity)	PO (once/twice daily)
Radezolid	No longer in development (unclear reason)	PO (once/twice daily)
Sutezolid	Phase 2 trials in TB ongoing	PO (once/twice daily)
TBI-223	Phase 2 trials ongoing	PO (once/twice daily)

This report aims to review the evidence for using linezolid and tedizolid to treat infections caused by staphylococcal species (including *Staphylococcus aureus* and CoNS). We describe adverse effects, drug interactions and contraindications for their use, and investigate the potential for treatment optimization. We anticipate that this article will provide support for those considering utilization of linezolid and tedizolid in clinical practice.

This expert-led narrative review was developed collaboratively by clinicians and pharmacists within the ESGS. Relevant literature was identified iteratively through PubMed searches, review of reference lists and the authors’ knowledge of the literature. A prespecified systematic search strategy was not used. Evidence was selected according to its relevance to the pharmacology, antimicrobial activity, and clinical use and safety of linezolid and tedizolid in the management of staphylococcal infections.

## Mechanism of action and antimicrobial spectrum

Tedizolid is typically more potent than linezolid, allowing for once-daily dosing, compared with twice-daily dosing for linezolid. The structure of tedizolid, which is ingested as a prodrug and converted to an active form, has a modified side chain to improve bacterial susceptibility and an optimized C- and D-ring system to improve potency through binding site interactions.^2^ Tedizolid also maintains some activity against linezolid-resistant bacterial strains due to structural differences that mitigate mechanisms of cross-resistance.

Oxazolidinones have distinct chemical structures and mechanisms of action (Figure 1). Both linezolid and tedizolid are bacteriostatic and work by inhibiting bacterial protein synthesis. Specifically, they bind to the A site of the 23S peptidyl transferase centre (PTC) within the 50S ribosomal subunit. The PTC is a critical region responsible for catalysing peptide bond formation during protein synthesis. By occupying the A site, oxazolidinones obstruct the normal function of this centre, preventing the binding of mRNA and tRNA to the ribosomal subunits. This binding interferes with the formation of the 70S initiation complex, which is essential for protein synthesis initiation and effectively stops early-stage protein synthesis. The oxazolidinone binding site overlaps with that of several other peptidyl transferase inhibitors such as chloramphenicol, clindamycin and macrolides. However, its interactions with the ribosome are specific enough to allow it to maintain its activity against bacterial pathogens that have developed resistance to other antibiotics targeting the ribosome. This specificity ensures the continued effectiveness of these antimicrobials in treating infections caused by drug-resistant bacterial strains.^3^

**Figure 1.**
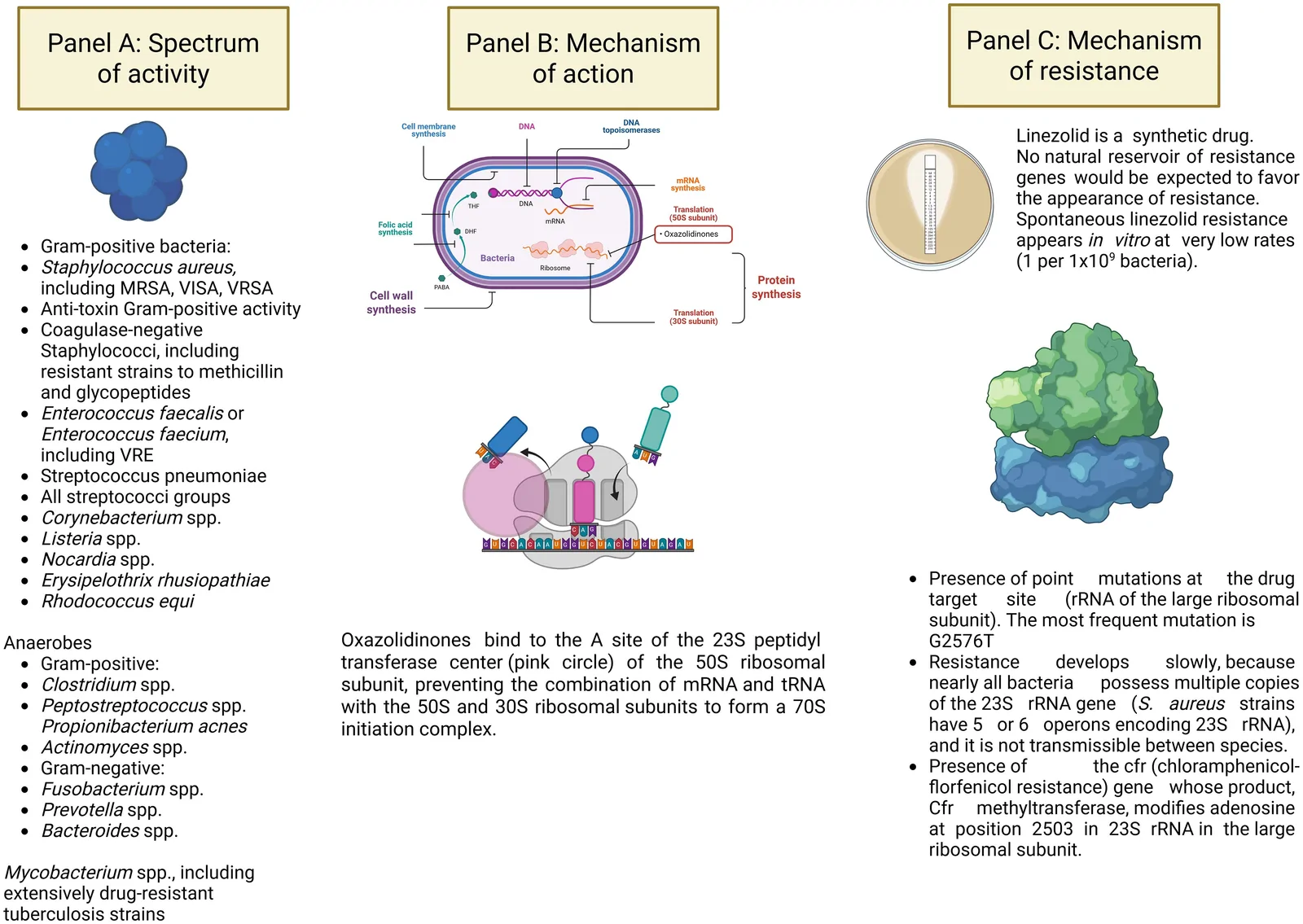
Spectrum of antimicrobial activity, mechanism of action, and mechanisms of resistance of linezolid and tedizolid. Created in BioRender. Del Pozo León, J. L. (2026) https://BioRender.com/mwkdrxo. DHF, dihydrofolate; PABA, para-aminobenzoic acid; THF, tetrahydrofolate; VISA, vancomycin-intermediate *Staphylococcus aureus*; VRSA, vancomycin-resistant *Staphylococcus aureus*.

## Antimicrobial resistance

Surveillance data show that resistance of staphylococcal species to linezolid and tedizolid remains low (<1% for most settings), although it is increasing globally. For linezolid, data from systematic reviews reported resistance rates for *S. aureus* of ∼0.05% (13/23 077 isolates),^4^ and surveillance data of clinical isolates from Germany and the USA reported resistance rates of 0.4% (10/2848) and 0.1% (∼3/3031), respectively.^5,6^ A review by Gu *et al.* in 2013 reported rates of resistance for CoNS of 1.4% (73/5202),^4^ although a more recent report indicates that linezolid resistance is emerging and local epidemiological data should be considered.^7^ There are limited clinical data on resistance rates for tedizolid, partly due to limited susceptibility testing. Data from the Surveillance of Tedizolid Activity and Resistance (STAR) programme across the United States and Europe between 2009 and 2013 showed that tedizolid inhibited 99.7% of all isolates (*n* = 11 231) tested: 99.9% of MSSA (*n* = 4579), 99.6% of MRSA (*n* = 3234) and 99% of CoNS (*n* = 623).^8^ More recent data (2015–2019) from this same surveillance programme found 99.9% of 35 978 *S. aureus* isolates to be susceptible to tedizolid, regardless of methicillin susceptibility.^9^

In clinical isolates, the most frequent mechanism of resistance is attributed to mutations at the site of action for the drug, mainly in the rRNA of the large ribosomal subunit. For example, point mutations in the 23S rRNA gene alter the ribosomal binding site, reducing linezolid affinity. Resistance develops gradually, partly due to the presence of multiple copies of the 23S rRNA gene in most bacteria, with *S. aureus* strains possessing 5–6 operons encoding 23S rRNA.^10^ Other reported mechanisms of linezolid resistance include point mutations in ribosomal proteins L3, L4 and L22; horizontal acquisition of a plasmid-mediated chloramphenicol-florfenicol resistance (*cfr*) gene that encodes a methyltransferase that modifies 23S rRNA and reduces susceptibility to linezolid and other ribosome-targeting antimicrobials;^11^ and acquisition of the ABC transporter genes known as *optrA* and *poxtA*, which also mediate linezolid resistance.^12,13^ Mechanisms of resistance to tedizolid are broadly similar to those for linezolid. However, tedizolid generally retains potent *in vitro* activity against *cfr*-positive VRE and MRSA isolates, likely due to structural differences that reduce the impact of *cfr*-mediated ribosomal methylation.^14,15^ This observation is based primarily on *in vitro* susceptibility data from a limited number of *cfr*-positive isolates, and whether this retained *in vitro* activity translates into clinical efficacy remains uncertain.

## Key evidence for the use of linezolid in staphylococcal disease

There is robust evidence from randomized controlled trials (RCTs) to support the use of linezolid as an effective treatment option for staphylococcal infections, particularly those caused by MRSA. Key advantages also include oral administration with high bioavailability, making it a suitable choice for outpatients and as an oral step-down. However, close monitoring for potential adverse effects is needed, particularly where prolonged treatment (>14 days) is needed.

In 2008 a meta-analysis published in *The Lancet* reviewed 12 RCTs including over 6000 participants to clarify whether linezolid is superior to glycopeptides or β-lactams for the treatment of Gram-positive infections.^16^ Overall, it was found that linezolid was more effective than glycopeptides and β-lactams with respect to treatment success (OR 1.41; 95% CI, 1.11–1.81) but mortality was similar between groups. This group found that linezolid was more effective than comparators for those with skin and soft tissue infections and bacteraemia, but that there was no significant difference for treating pneumonia. These findings should be interpreted with caution due to significant heterogeneity between the trials, which encompassed different Gram-positive infections (as opposed to just staphylococcal), infection syndromes, patient populations and comparator antibiotics. Furthermore, many of the studies pre-dated current vancomycin dosing and therapeutic drug monitoring, and the vancomycin exposure in the historical comparator groups may not reflect or be transferrable to current practice. Treatment with linezolid was not associated with more adverse effects in general (OR 1.40; 95% CI, 0.95–2.06); however, thrombocytopenia was recorded more commonly in patients receiving linezolid (OR 11.72; 95% CI, 3.66–37.57). One of the RCTs included in this meta-analysis compared the efficacy of linezolid with that of vancomycin in the treatment of suspected or proven methicillin-resistant Gram-positive complicated skin-and-soft tissue infections (CSSTIs) requiring hospitalization. They found that in the intent-to-treat population, 92.2% and 88.5% of patients treated with linezolid and vancomycin, respectively, were clinically cured at the test-of-cure visit (*P* = 0.057), and that linezolid outcomes were superior to vancomycin outcomes for patients with MRSA infections (*P* < 0.001).^17^ A later RCT in 2012 enrolled 348 patients with MRSA pneumonia and randomized patients to either IV linezolid (600 mg 12-hourly) or IV vancomycin (15 mg/kg 12-hourly) for 7–14 days (or 21 days in the case of associated bacteraemia).^18^ In the per protocol population, 95/165 (58%) in the linezolid arm and 81/174 (46.5%) in the vancomycin arm achieved the primary outcome of clinical success at end of study (*P* = 0.042). Clinical response for the treatment of MRSA nosocomial pneumonia was significantly higher for those treated with linezolid than with vancomycin, although 60 day mortality was similar.^18^ Although these data appear to contradict the results from the meta-analysis published earlier in 2008, which did not show a significant difference for treatment of pneumonia, this study focused on MRSA pneumonia rather than mixed Gram-positive infections. These findings are supported by international guidance; the IDSA guidelines recommend linezolid as an effective alternative to vancomycin for treating MRSA pneumonia and CSSTIs.^19^

Lastly, there is an increasing body of evidence—mainly case reports—to support using linezolid to treat infections caused by susceptible CoNS including CNS infections (ventriculitis), endocarditis and prosthetic joint infections.^20,21^

## Key evidence for the use of tedizolid in staphylococcal disease

The two main RCTs (ESTABLISH-1 and ESTABLISH-2) show that tedizolid is non-inferior to linezolid for the treatment of ABSSSIs. ESTABLISH-1 is a randomized double-blind trial that aimed to establish the non-inferiority of tedizolid (200 mg once daily for 6 days) versus linezolid (600 mg twice daily for 10 days) for treatment of ABSSSIs, and to compare their safety. This study included 667 participants, 346 of whom had proven staphylococcal infection (178 MRSA and 170 MSSA).^22^ Tedizolid was statistically non-inferior to linezolid, and clinical response rates were high. Adverse event rates were similar for both groups, with fewer gastrointestinal adverse events among patients treated with tedizolid. Although the relatively shorter duration of tedizolid demonstrated non-inferiority, the differences in treatment duration should be considered when interpreting comparisons of adverse events. For example, thrombocytopenia was more frequent in the linezolid group, but this may reflect the longer treatment duration in this group as platelet suppression is common at Days 7–10. Whether this apparent haematological tolerability advantage of tedizolid persists when the two agents are administered for equivalent, longer durations remains uncertain. This is particularly relevant in clinical practice where treatment of deep-seated staphylococcal infections may extend beyond 10 days and it remains an important knowledge gap. Additionally, the study was not adequately powered to make conclusions about the risk of myelosuppression. ESTABLISH-2 was a randomized double-blind non-inferiority trial comparing IV then oral tedizolid (200 mg once daily for 6 days) with IV then oral linezolid (600 mg twice daily for 10 days) in adults with ABSSSI. It showed that tedizolid was non-inferior.^23^

Mensa *et al.*^24^ performed a study to assess the indications for and to describe the long-term safety profile of tedizolid. They reviewed 81 patients who had received tedizolid for a median of 28 days. Over 80% of participants had tedizolid selected to avoid potential linezolid toxicities or interactions, or due to previous linezolid-related toxicities, highlighting the potential role for this agent where linezolid is not tolerated. The review found that long-term tedizolid treatments had good tolerance, with rates of gastrointestinal adverse effects and haematological toxicity lower than those reported with linezolid, particularly in patients with chronic renal failure and in those with a history of linezolid-associated toxicity. The renal excretion of linezolid may provide a mechanistic explanation as to why tedizolid may be better tolerated in those with renal impairment. However, reducing the dose of linezolid, with therapeutic drug monitoring (TDM), may have enabled ongoing therapy.

Although clinical data supporting the use of tedizolid to treat CoNS infections are limited, *in vitro* studies demonstrate activity—including methicillin-resistant isolates—as discussed previously. There are also theoretical advantages for its use over linezolid including higher potency and potentially better tolerability. For example, a non-comparative open-label trial followed up 37 patients with confirmed bone and joint infections who had tedizolid for 12 weeks (mean) and found that it was well tolerated without significant toxicity.^25^

## Pharmacokinetics and pharmacodynamics

For both linezolid and tedizolid, antimicrobial efficacy is related to drug exposure relative to the susceptibility of the organism, with AUC/MIC representing an important pharmacokinetic/pharmacodynamic (PK/PD) index. For linezolid, an AUC_0–24_/MIC of approximately 80–120 has been associated with clinical and microbiological response.^26^ For tedizolid, free-drug AUC/MIC (fAUC/MIC) appears to be the principal PK/PD index.^27^ The standard dosage of linezolid is 600 mg every 12 h for adults, while tedizolid is administered at 200 mg every 24 h. This may be via oral or parenteral administration. Oral absorption is rapid. Pharmacodynamics are shown in Figure 2. Linezolid and tedizolid are well absorbed into the kidneys, lungs, bone and brain. This enables their use in a range of Gram-positive infections such as pneumonia and osteomyelitis. These data are briefly summarized in Table 2.

**Figure 2.**
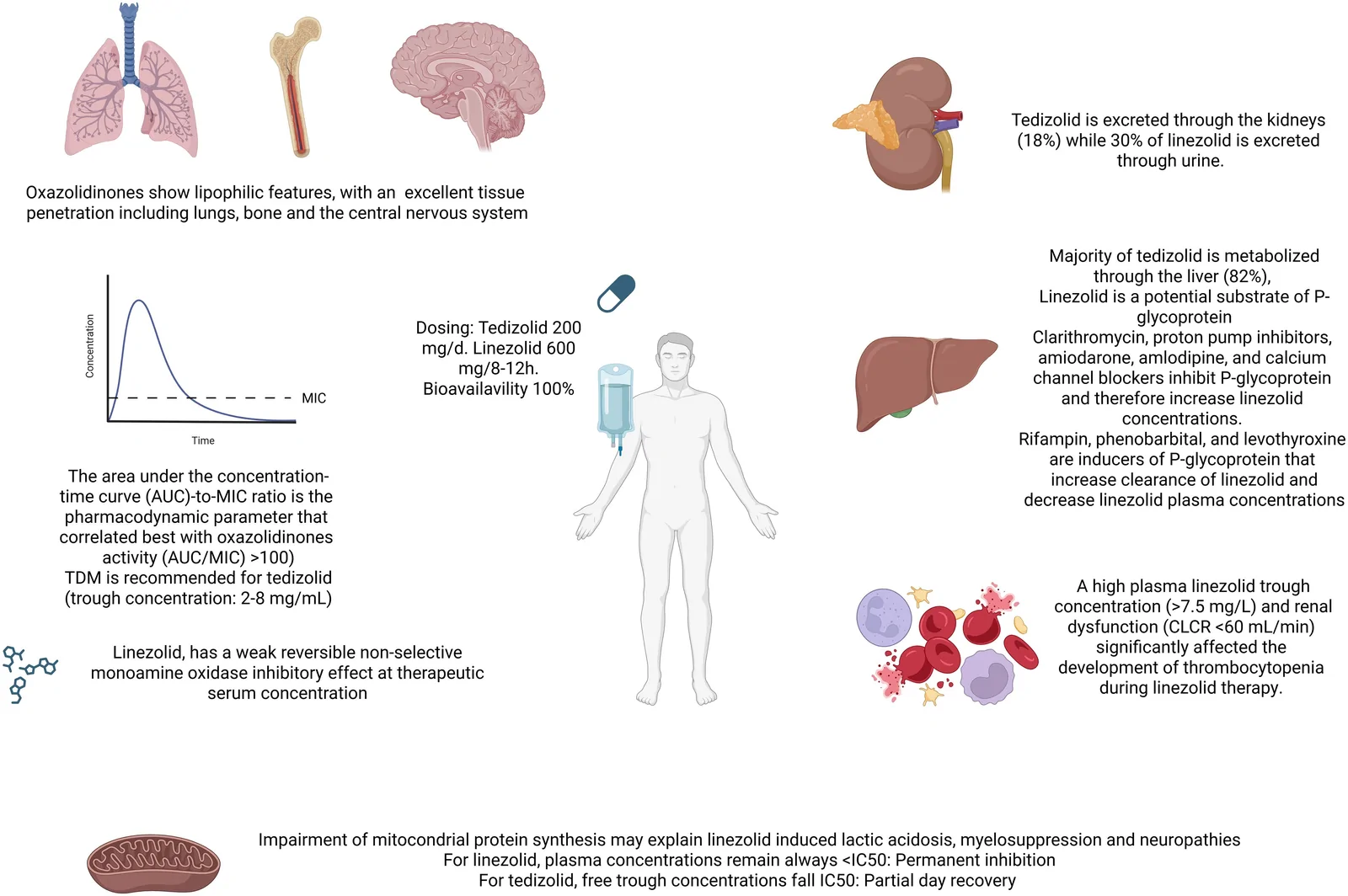
Pharmacokinetic and pharmacodynamic properties of linezolid and tedizolid: tissue distribution, metabolism, drug interactions, and toxicity. Created in BioRender. Del Pozo León, J. L. (2026) https://BioRender.com/x2ohgq7. AUC, area under the curve; CLCR, creatinine clearance; MIC, minimum inhibitory concentration.

**Table 2.** Pharmacokinetic properties of linezolid and tedizolid

Drug	Study population	Dosing	*t*_1/2_, h	PK/PD target for efficacy	AUC, mg h/L	*C*_max_, mg/L	*C*_min_, mg/L	MIC, mg/L
Linezolid	Healthy volunteers	Oral 600 mg, 12-hourly	3.5–7	*T* > MIC target of ≥85% or AUC/MIC >100	107–138	16.3–21	2–7	4
	ITU	IV 600 mg, 12-hourly	5.52 ± 3.63	*T* > MIC of >85%, AUC/MIC 80–120 Achieved cure in 4/8 of ITU patients	96.73 ± 56.45	15.70 ± 6.58 (at 1.4 h)	4.10 ± 4.99 (at 12 h)	2
Tedizolid	Healthy volunteers	Oral 200 mg, 24-hourly	8.9 ± 2.2	*f*AUC/MIC ≥3	5.2 ± 1.6	2.7 ± 1.1 (at 2–3 h)	—	0.5

*f*AUC, bioavailability-free drug area under the curve; ICU, intensive care unit; *T* > MIC, time above minimum inhibitory concentration.

Data reported as median ± SD or range.

Tedizolid undergoes hepatic metabolism, with approximately 82% of the drug being processed in the liver. For linezolid, about 30% of its elimination occurs via the urine. This indicates that linezolid may be preferable for patients with severe liver impairment^28^ and that different routes of elimination have important implications for clinical practice.^29^

Adequate exposure to linezolid is generally accepted as *C*_min_ of 2–8 mg/L.^30,31^ In a study by Song *et al.*,^32^ mean linezolid trough concentrations were correlated with risk for adverse events. Elevated plasma linezolid concentrations, above 8 mg/L, and the presence of renal dysfunction (creatine clearance <60 mL/min) have been observed to have a substantial impact on the development of thrombocytopenia.^33,34^ When long courses (>28 days) are planned, for critically unwell patients, or those with renal dysfunction, TDM may help avoid toxicity but can be costly.^35^ There are no well-established clinical target ranges and no validated therapeutic trough ranges for tedizolid. However, the predominant hepatic metabolism and the fact that it is not considered a cytochrome P450 (CYP) substrate indicates that tedizolid may require a different approach with less regular TDM.

Linezolid exposure may be unpredictable in critically ill patients or in cases with organ dysfunction, renal replacement therapy and augmented renal clearance. Expert guidance recommends TDM in critically ill patients receiving linezolid but comparable data for tedizolid are limited, and there is currently insufficient evidence to recommend routine TDM or dose modification.^36,37^

Standard doses of linezolid may also be inadequate for obese patients.^38^ A recent systematic review exploring the pharmacokinetics of antibiotics suggested that patients with obesity and preserved renal function might require higher dosing of linezolid, although the certainty of evidence was low.^39^ In contrast, available pharmacokinetic data suggest that dose adjustment of tedizolid is not routinely required in obesity. Population pharmacokinetic modelling found no clinically significant effect of body weight or BMI on tedizolid exposure, while a dedicated pharmacokinetic study comparing nine morbidly obese adults with nine matched non-obese controls found no significant differences in AUC, *C*_max_, clearance or half-life following a 200 mg IV dose, supporting use of the standard 200 mg once-daily regimen in obesity.^27,40^ These data are based on relatively small numbers of patients with severe obesity and should therefore be interpreted cautiously.

A history of Roux-en-Y gastric bypass does not appear to affect dosing for either agent.^41^

A consensus statement has been produced to discuss the importance of therapeutic monitoring and personalization of linezolid dosing. It highlights the need to consider factors such as disease severity and obesity when determining dosing to optimize the efficacy and safety of treatment in a variety of patients.^36^

## Safety

### Drug interactions

Linezolid is a reversible, non-selective inhibitor of monoamine oxidase, an enzyme responsible for breaking down serotonin in the CNS. When combined with other serotonergic medications, it can increase serotonin levels and precipitate serotonin syndrome (SS). This concern has led to recommendations for avoidance and cautious use in patients receiving serotonergic agents.^42^ The summaries of product characteristics (SmPCs) for both linezolid and tedizolid report the potential for interaction with adrenergic and serotonergic agents with caution advised.^43,44^ This regulatory recommendation poses a clinical challenge when immediate treatment of serious infections is necessary. However, the absolute risk of SS is low and most reported cases of SS involve multiple agents, prolonged overlap and high dose/more potent serotonergic drugs. A systematic review of 84 studies reported incidences of SS of 0.005% with linezolid monotherapy and 0.0134% when co-administered with other serotonergic agents.^45^

A large study reviewed 2208 participants who were taking serotonergic agents concomitantly with linezolid across 20 antibiotic trials.^46^ The authors did not find a significant difference in serotonin toxicity between those on linezolid and those on other antibiotics. The rates of serotonin toxicity were low in both groups: 9 of the 2208 (0.41%) linezolid patients and 3 of the 2057 (0.15%) patients who were taking other antibiotics. Definitions of a ‘serotonergic agent’ in this study were variable, including those on analgesics such as codeine, and antiemetics such as metoclopramide. There were over 500 patients per group taking antidepressant medication, such as a selective serotonin reuptake inhibitor (SSRI) or tricyclic antidepressant, but very few were taking a monoamine oxidase inhibitor (MAOI) (three in the linezolid arm and none in the comparator arm) reflecting their low usage in practice. Gatti *et al.*^47^ evaluated 669 FDA post-marketing reports of SS mentioning linezolid, and suggested that linezolid plus citalopram, escitalopram or methadone may be more likely to cause SS than other drug combinations, related to their pharmacological properties including lipophilicity, large volume of distribution and potent serotonin reuptake transporter inhibition.^48^ Recent observational studies reviewed the records of 1743 and 1134 patients taking both linezolid and serotonergic agents and reported low rates of possible SS (<0.11% and <0.5%, respectively).^49,50^ Tedizolid inhibits MAO less potently *in vitro* and did not produce significant pressor/serotonergic effects in human or animal models.^51^ A retrospective observational cohort study of 479 adult patients receiving tedizolid, of whom 62% (297) were on concomitant serotonergic agents, reported that possible SS requiring cessation of tedizolid occurred in 0.4% of cases (2/479).^52^ However, real-world data on tedizolid use with serotonergic drugs are still limited.

It is prudent to avoid both linezolid and tedizolid in those taking MAOIs, unless the MAOI may be discontinued, and it is possible to wait for 2 weeks before commencing an oxazolidinone. However, SS is rare and the risk/benefit for linezolid use in patients receiving other serotonergic drugs can be acceptable; factors including potency of the serotonergic agent and duration of treatment should be considered. One may consider giving an SSRI with linezolid or perhaps tedizolid, but patients and their clinicians should be informed of the SmPC guidance for caution and risk of serotonin toxicity to enable early recognition and cessation of the causative agent in the case of such an event.^48^

Linezolid has been identified as a potential substrate of P-glycoprotein, a transport protein in the cell membrane. Certain drugs, such as clarithromycin, proton pump inhibitors, amiodarone and calcium channel blockers, exert an inhibitory effect on P-glycoprotein.^53^ As a result, these substances increase blood concentrations of linezolid, which may increase the risk of adverse effects; however, the clinical significance remains uncertain and routine empirical linezolid dose reduction is not recommended solely on this basis. On the other hand, drugs such as rifampicin, phenobarbital and levothyroxine act as inducers of P-glycoprotein, which accelerates the elimination of linezolid and decreases its plasma concentrations. These drug interactions highlight the importance of careful assessment of concomitant medications and consideration of adjusted dosing in patients receiving linezolid or related drugs.^42^

Clindamycin also interacts with linezolid to increase the blood levels of linezolid. If these two agents are prescribed together to act against bacterial toxins, this may exacerbate linezolid side effects.

### Haematological toxicity and renal impairment

Weekly monitoring of haematological parameters is recommended in patients treated with linezolid, due to the potential for thrombocytopenia and myelosuppression.^33,54^ Due to its renal excretion, dose reduction may be sensible for those with renal impairment, and dose adjustment to e.g. 300 mg twice a day can be considered in those with reduced creatinine clearance.^48^ As discussed, TDM may help to avoid toxicity. In contrast tedizolid is not excreted via the kidneys so could be considered in cases of renal impairment.

### Linezolid-induced lactic acidosis

Prolonged treatment with linezolid (>28 days) has been associated with acidosis, perhaps via mitochondrial mechanisms and lactic acidosis.^55^ This finding sheds light on the possible underlying mechanisms of linezolid-associated adverse effects, highlighting the importance of understanding the relationship between drug activity and mitochondrial function.^56^ The UK SmPC does not specifically recommend routine lactate monitoring;^44^ lactate should be measured promptly at any treatment duration in patients who develop unexplained nausea, vomiting, abdominal pain, low serum bicarbonate or hyperventilation. Lactic acidosis has not been reported with tedizolid when used for short periods, but when prolonged courses have been given, it has occurred.^57^

### Impact of linezolid and tedizolid on mitochondrial protein synthesis and its reversibility

Oxazolidinones can inhibit mitochondrial protein synthesis in human cells because of structural similarities between bacterial and mitochondrial ribosomes. This effect is clinically important because mitochondrial function provides a mechanistic explanation for several associated toxicities including lactic acidosis, myelosuppression and neuropathies. Differences in pharmacokinetic exposure may influence the mitochondrial toxicity of individual oxazolidinones. Tedizolid is a more potent inhibitor of mitochondrial protein synthesis *in vitro* than linezolid, however its once daily dosing results in a longer period during which unbound plasma concentrations fall below the mitochondrial protein synthesis IC₅₀ (50% inhibitory concentration).^28^ This could allow mitochondrial protein synthesis to recover between doses and could contribute to differences in toxicity during prolonged treatment, although this has not been confirmed clinically. Host genetic factors may also influence an individuals susceptibility to mitochondrial toxicity, patients with the A2706G mitochondrial polymorphism may be more susceptible to toxicity. Therefore it may be an option to screen patients for this mutation in the future to ensure safer use of these antibiotics.^55^

### Pregnancy

Linezolid and tedizolid have been assigned pregnancy category C by the FDA—animal studies have shown adverse effects on the foetus and there are no adequate, well-controlled studies in pregnant women. Both drugs have been found in breast milk.^58^

## Conclusions

Oxazolidinones are effective against Gram-positive bacteria, in particular organisms with drug resistance. They have high oral bioavailability, and their lipophilic characteristics give them excellent penetration into tissues. For linezolid, the main factors limiting its use are potential drug–drug interactions and its toxicity profile (especially in renal impairment). In some cases, standard doses of linezolid may not be suitable and TDM can be helpful. Preliminary results of preclinical and clinical pharmacology studies suggest that the unique mode of action of tedizolid improved PK/PD; lower doses and lack of monoamine oxidase inhibition *in vivo* may translate to improved safety compared with linezolid. However, tedizolid use is currently limited by its high cost.
